# A Functional 3′ UTR Polymorphism of *FADS2* Affects Cow Milk Composition through Modifying Mir-744 Binding

**DOI:** 10.3390/ani9121090

**Published:** 2019-12-06

**Authors:** Mingxun Li, Xubin Lu, Qisong Gao, Mengqi Wang, Abdelaziz Adam Idriss Arbab, Yujia Sun, Zhi Chen, Huimin Zhang, Niel A. Karrow, Zhangping Yang, Yongjiang Mao

**Affiliations:** 1 Key Laboratory of Animal Genetics & Breeding and Molecular Design of Jiangsu province, Yangzhou University, Yangzhou 225002, China; limingxun@live.com (M.L.); xubinlu@139.com (X.L.); 18305182715@163.com (Q.G.); mengqi.wang.1@ulaval.ca (M.W.); arbabtor@yahoo.com (A.A.I.A.); chenzhijerom@163.com (Z.C.); minmin-911@163.com (H.Z.); yzp@yzu.edu.cn (Z.Y.); 2 Joint International Research Laboratory of Agriculture and Agri-Product Safety of Ministry of Education of China, Yangzhou University, Yangzhou 225002, China; ysunshine30@outlook.com; 3 Center for Genetic Improvement of Livestock, Department of Animal Biosciences, University of Guelph, Guelph, ON N1G 2W1, Canada; nkarrow@uoguelph.ca

**Keywords:** fatty acid desaturase 2, functional mutation, miR-744, milk fatty acids, Chinese Holstein cows

## Abstract

**Simple Summary:**

Fatty acid desaturase 2 (*FADS2*) is the rate-limiting enzyme involved in the synthesis of long-chain polyunsaturated fatty acids (LC-PUFAs). Many studies have suggested that polymorphisms in the *FADS2* gene can modify its delta-6 desaturase activity. However, much remains unknown in regards to the regulatory mechanisms interpreting how DNA variants influence the function of *FADS2*. A previous study has suggested that c.1571G>A is located within the miR-744 binding site, indicating that this single nucleotide polymorphism (SNP) may be functional. Therefore, the aim of the present study was to determine the association of SNP c.1571G>A with milk PUFAs content and to further elucidate how this SNP contributes to regulating *FADS2* expression. Our results support the use of SNP c.1571G>A as a potential genetic marker in the selective breeding of cattle to increase beneficial FAs content in milk.

**Abstract:**

This study determined the associations of *FADS2* c.1571G>A with milk FAs content and revealed that cows with the GG genotype had improved levels of delta-6 desaturase substrates (linoleic acid, C18:2n-6; *p* < 0.001) and decreased levels of desaturase products (gamma-linolenic acid, C18:3n-6; *p* < 0.001), indicating a reduction in *FADS2* expression or delta-6 desaturase activity caused by this polymorphism. Computer alignment demonstrated that c.1571G>A occurred within a potential miR-744 binding site. When the c.1571G allele was present, the luciferase activity of reporter constructs was significantly suppressed by miR-744, while no such effect was observed with the A allele. Overexpression of miR-744 in bovine mammary epithelial cells (with the 1571GG genotype) downregulated *FADS2* expression at both mRNA and protein levels. In contrast, inhibition of endogenous miR-744 with a specific inhibitor dramatically upregulated *FADS2* expression. Taken together, these lines of evidence indicated that the c.1571A minor allele abolished the ability of miR-744 to bind *FADS2*, with a consequent increase in *FADS2* expression levels and synthesis of omega-6 LC-PUFAs.

## 1. Introduction

Advances in genomic information and modern analytical techniques have revolutionized the dairy cattle industry and have provided a new opportunity to identify specific genomic regions underlying complex phenotypic traits [1,2]. Association studies taking advantage of candidate-gene strategies are commonly used methods for probing the relationship between genetic variations in specific genomic regions and the underlying phenotypes of interest. In recent decades, a series of genes impacting important economic traits in dairy cattle have been located within quantitative trait loci (QTLs) scattered across the genome [2,3,4]. The integration of these QTLs into genetic assessment provides enormous potential to improve the efficiency and precision of cattle breeding, thus accelerating the genetic improvement of important economic traits.

Milk fat is one of the most important economic traits for dairy cattle milk. Although concerns have been expressed regarding the high amount of saturated fatty acids (SFAs) in whole milk, accumulating evidence demonstrates the presence of healthful fatty acids (FAs), such as long-chain polyunsaturated fatty acids (LC-PUFAs), which have been implicated in decreasing the risk of many diseases, such as neurodegenerative disorders, autoimmune responses, diabetes, and cardiovascular disease [5,6,7]. Therefore, in recent years, market attention has increasingly focused on increasing the healthfulness of milk fat, and thus the possibilities of modifying milk fat components by dietary or genetic means are getting more research attention [8]. However, the genetic approach is more feasible than dietary manipulation because of the influence of ruminal biohydrogenation, which can convert unsaturated fatty acids to more saturated end products [9]. This view is also supported by the moderate to high heritabilities of milk FAs [8,10,11].

The metabolism of LC-PUFAs is controlled through a series of elongation and desaturation of the fatty-acid molecules. The fatty acid desaturase 2 (*FADS2*) gene, which catalyzes the introduction of double bonds after the sixth carbon atom from the carboxyl end of the carbon chain, is the rate-limiting enzyme in the biosynthesis of LC-PUFAs [12,13]. The importance of *FADS2* in the synthesis of LC-PUFAs has been widely investigated in mice [14,15]. Stoffel et al. reported that deletion of *FADS2* hindered the conversion of linoleic acid (LA, C18:2n-6) to gamma-linolenic acid (GLA, C18:3n-6), which is the first step of the enzymatic cascade of omega-6 LC-PUFA synthesis, and revealed that *FADS2* was the only desaturase that catalyzes this critical step [14]. Stroud et al. demonstrated that *FADS2* null mice manifested a range of pathological features, such as hypogonadism, sterility, spleen and liver enlargement, dermatitis, and duodenum ulcers [15]. However, the regulatory mechanisms of *FADS2* expression have been scarcely explored.

LC-PUFAs present in dairy cattle milk have demonstrated many health benefits in humans. Recently, several studies revealed strong associations between single nucleotide polymorphisms (SNPs) in *FADS2* and altered delta-6 desaturase activities (D6D) which eventually contribute to the variability of endogenous FAs composition [16,17,18,19]. Polymorphisms in the promoter CpG islands of the *FADS2* gene are demonstrated to be closely correlated to the levels of omega-6 fatty acid arachidonic acid (ARA, C20:4n-6), as well as its precursors LA and GLA, in human serum phospholipids [16,17]. In cattle, Ibeagha-Awemu et al. reported the genetic diversity of the *FADS2* gene and analyzed the effects of identified SNPs on omega-6 and omega-3 milk FAs profiles in Canadian Holstein cows [20]. SNP c.1571G>A in the 3′ untranslated region (UTR) of *FADS2* has been associated with milk omega-6 FAs, C18:2n10t12c and C18:2n6tt, with genotype GG showing higher increases in the affected FAs before false discovery rate (FDR) correction [20]. Bioinformatics analyses suggested that c.1571G>A is located within the miR-744 binding site, indicating that this SNP may be functional [20]. However, much remains unknown in regard to the regulatory mechanisms explaining how this SNP influences the function of *FADS2*. Therefore, the aim of this study was to determine the association of SNP c.1571 G>A with milk PUFAs content in Chinese Holstein cows, and to further elucidate how this SNP contributes to regulating *FADS2* expression.

## 2. Materials and Methods

### 2.1. Milk Sample Collection and Fatty Acids Analysis

All animal experiments were carried out in accordance with the guidelines of Institutional Administrative Committee and Ethics Committee of Laboratory Animals (license number: SYXK [Su] 2017-0044) and were approved by the Yangzhou University Institutional Animal Care and Use Committee.

Milk samples were collected once per cow during the morning milking from 300 unrelated lactating Chinese Holstein cows in the experimental farm of Yangzhou University, Jiangsu, China. Cows in second or third lactation were selected to avoid age effect on the parameters to be estimated. After collection, samples were immediately transported in iceboxes to the laboratory. Somatic cell count (SCC) was determined within 24 h after collection of milk samples using a Fossomatic cell counter (Foss Electric, Hillerød, Denmark). Twenty-five cows with a milk SCC of ≥200,000 cells/mL were excluded from the analysis.

Milk FAs extraction and subsequent fatty acid methyl esters were conducted according to the Chinese national standard methods (GB 5413.27-2010). Briefly, the total FAs of 1 g milk were extracted with petroleum ether by Soxhlet extraction. After evaporating the solvent using a rotary evaporator under vacuum, 1 mL of 10% pyrogallic acid methanol was added into the flask containing the fat concentrate, and the samples was evaporated to dryness in a 65 °C water bath. Then, 10 mL of 0.5 mol/L KOH-methanol was added and refluxed for 5–10 min at 80 °C. Next, 5 mL of 14% BF3-MeOH was added and refluxing was continued for an additional 15 min. After cooling, the mixture was transferred to a new 50 mL centrifugal tube and washed 3 times with 3 mL of saturated NaCl solution. The washing liquid was then transferred to the 50 mL centrifugal tube and 10 mL hexane was added, and then the mixture was oscillated and centrifuged at 5000× *g* for 5 min. The supernatant containing FA methyl esters were collected for gas chromatography (GC) analysis.

Fatty acid methyl esters were measured using an Agilent 7890A gas chromatograph coupled to an Agilent 5975C inert mass-selective detector, equipped with an Agilent 7693 autosampler and an Agilent DB23 column (60 m length × 0.25 mm internal diameter × 0.15 µm film thickness). The flow rate of nitrogen carrier gas was 1.0 mL/min. The injector was set at 260 °C with a split ratio of 30:1 and the detector was set at constant 280 °C. The temperature of the gas chromatograph began at 140 °C and was held for 5 min, increased at 4 °C/min to 240 °C, and held for 15 min. Individual FAs were identified by comparison retention times with fatty acids standards (Nu-Chek-Prep, GLC-NESTLE-37, Elysian, MN, USA). Concentrations of 23 identified FAs were expressed as weight-proportion of total fat weight (w/w%). As additional traits for association studies, concentrations of SFA, MUFA, and PUFA, C18 index, and delta-6 desaturase activities (D6D index, C18:3n-6/C18:2n-6) were calculated using data for all 23 identified milk FAs. Descriptive statistics for milk fatty acids composition are given in Table S1.

### 2.2. Blood Sampling, DNA Extraction, SNP Discovery, and Genotyping

Blood samples were collected from the above-mentioned 275 Chinese Holstein cows. Genomic DNA was extracted from the white blood cells using a TIANamp Blood DNA Kit (Tiangen, Beijing, China), following the manufacturer’s instructions. DNA concentrations were measured with the Nanodrop ND-1000 spectrophotometer (Thermo Fisher Scientific, Waltham, MA, USA) and evaluated for integrity by 1% agarose gel electrophoresis.

DNA samples from 20 randomly selected cows were utilized to amplify and sequence the 3′ UTR of *FADS2* using primers designed with Primer3 software to detect SNP [21]. The PCR reactions were carried out in a PTC-200 DNA Engine Cycler (Bio-Rad, Hercules, CA, USA) using an optimal annealing temperature (Table S2) determined by a PCR temperature gradient. Twenty microliters of PCR amplicons were sent to Shanghai Sangon Company (Shanghai, China) for Sanger sequencing using an ABI PRISM 3700 DNA Sequencer (Applied Biosystems, Foster, CA, USA). After sequencing, the forward and reverse sequences were assembled using ContigExpress module in Vector NTI Advance 11.5 (Invitrogen, Carlsbad, CA, USA) to discover novel SNP. Animal genotyping for the discovered SNP was carried out with Sequenom MassARRAY platform using 10 ng of genomic DNA dissolved in DNase-free water [22].

### 2.3. Cell Cultures

The original bovine mammary epithelial cells (MAC-T) were purchased at Nexia Biotechnologies (Quebec, QC, Canada). Human embryonic kidney 293 transformed cells (HEK293T) were purchased from The Cell Bank of Type Culture Collection of Chinese Academy of Sciences (Shanghai, China). MAC-T cells and HEK293T cells were cultured in Dulbecco’s modified Eagle’s medium supplemented with 10% fetal bovine serum (FBS, Gibco, Carlsbad, CA, USA), and 100 U/mL penicillin/streptomycin (Sigma-Aldrich, Shanghai, China) at 37 °C in a humidified atmosphere with 5% CO_2_.

### 2.4. In Silico miRNA-Target Interaction Analysis and Luciferase Reporter Assay

RNA22 [23], RNAhybrid [24], and TargetScan Release 7.2 [25] were used to predict the effects of c.1571 G>A polymorphism on possible miRNA binding sites. SNP c.1571G>A was found to alter a binding site for miR-744. Mechanistic interactions between miR-744 and *FADS2* 3′ UTR were confirmed experimentally by luciferase reporter assay. Two oligo variants, *FADS2-1571G* and *FADS2-1571A*, containing the polymorphic site and miR-744 binding site, were directly synthesized (from +1421 to +1720 relative to the transcription start site) and then cloned into pmirGLO dual luciferase reporter plasmids (Promega, Madison, WI, USA) using the restriction enzymes *Sac*I and *Xho*I. The resulting constructs were designated pmirGLO-1571G and pmirGLO-1571A, respectively. The sequences of the constructs were verified by DNA sequencing.

For the luciferase assay, MAC-T and HEK293T cell lines were seeded in a 24-well culture plate at a density of 1–2 × 10^5^ cells per well. After 24 h, miRNA-744 mimic or mimic controls (GenePharma, Shanghai, China) were cotransfected with the reporter vectors (pmirGLO-1571G or pmirGLO-1571A allele constructs) using Lipofectamine 2000 (Thermo Fisher Scientific, Carlsbad, CA, USA) in triplicate. Forty-eight hours after transfection, 10 μL of cell lysate were collected to measure the activities of Firefly and Renilla luciferases using the Dual-Luciferase Reporter Assay System (Promega) with a Multilabel Counter luminometer (Varioskan Flash, Thermo Fisher Scientific). The Firefly luciferase activity was determined first by adding 50 μL LAR II to create a luminescent signal. The reaction was quenched by adding 50 μL Stop & Glo Reagent, and the Renilla luciferase activity was then measured. The relative luciferase activity was acquired by normalizing Firefly luciferase activity to Renilla luciferase activity. The results were further normalized to the negative miRNA control. All assays were performed in triplicate and repeated three times. A Student’s *t*-test was used to compare two different groups and a *p* ≤ 0.05 was considered to be significant.

### 2.5. Reverse Transcription-Quantitative Polymerase Chain Reaction (RT-qPCR)

Total RNAs were extracted from MAC-T cells using RNAiso Plus (Takara, Dalian, China) according to the manufacturer′s instructions. The RNA quantity and purity were measured using the NanoDrop 1000 (Thermo Fisher Scientific). The 260/280 and A 260/230 ratios were 1.96 ± 0.02 and 1.99 ± 0.17, respectively. For *FADS2* expression assay, the complementary DNA was synthesized using the PrimeScript RT Reagent Kit with gDNA Eraser (Takara). The RT-qPCR was performed on an ABI Prism 7300 sequence detection system (Applied Biosystems) using the TB Green Premix Ex × Taq II kit (Takara). *ACTB* and *GAPDH* were used as endogenous controls. For miRNA expression assay, reverse transcription was performed using Mir-X miRNA First-Strand Synthesis Kit (Clontech). The RT-qPCR was conducted by Mir-X miRNA RT-qPCR TB Green Kit (Clontech) and *U6* was used as an endogenous control. Relative gene expression levels were quantified by the 2^−ΔΔCt^ method. Primers used in this study are listed in Table S2. Statistically significant differences between groups were calculated using Student’s *t*-test. A *p*-value of <0.05 was considered to be significant.

### 2.6. Western Blot

Total proteins were extracted from MAC-T cells using RIPA lysis buffer supplemented with PMSF (Solarbio, Beijing, China) and quantified with a BCA protein assay (Solarbio). Equal amounts of protein were separated on 10% SDS-PAGE gels. Primary antibodies against FADS2 (catalog #PA5-25285) were purchased from Thermo Fisher Scientific. Antibodies against *β*-actin (catalog #SC-47778) were obtained from Santa Cruz Biotechnology (Santa Cruz, CA, USA). The secondary HRP-conjugated goat anti-rabbit IgG antibody (catalog #31210) was purchased from Thermo Fisher Scientific. Signals were visualized with the ECL Plus kit (Solarbio) and imaged under the ChemiDoc XRS System (Bio-Rad, Hercules, CA, USA).

### 2.7. Statistical Analysis

Statistical analyses were performed with SPSS v20.0 (IBM, Chicago, IL, USA) and Graphpad Prism 7.0 (GraphPad Software, La Jolla, CA, USA). The allele frequency, genotype frequency, and Hardy–Weinberg equilibrium (χ^2^ test) were calculated directly. Associations between *FADS2* genotypes and different FAs profiles were tested with the following mixed linear model:

y*_ijkl_* = μ + M*_i_* + P*_j_* + G*_k_* + e*_ijkl_* (1)

where y*_ijkl_* is the phenotypic record for the analyzed trait; μ is the overall mean; M*_i_* is the fixed effect of the *i*th stage of lactation (*i* = 1 to 10, 10 levels of 30 d each); P*_j_* is the fixed effect of the *j*th parity of the cow (*j* = 2 to 3); G*_k_* is the fixed effect of genotype (*j * =  3); and e*_ijkl_* is the random residual.

Allelic substitution effect (*α*) was calculated according to Falconer and Mackay (1996) [26]: *α* = *a* + *d*(*q* − *p*), where *a* is the additive effect and *d* is the dominant effect; *q* is the frequency of minor allele, and *p* is the frequency of major allele. Statistical significance of the genotype effects was determined using one-way analysis of variance followed by Bonferroni’s post-test.

## 3. Results

### 3.1. Identification of SNPs in the 3′ Untranslated Region of FADS2

**Figure 1:**
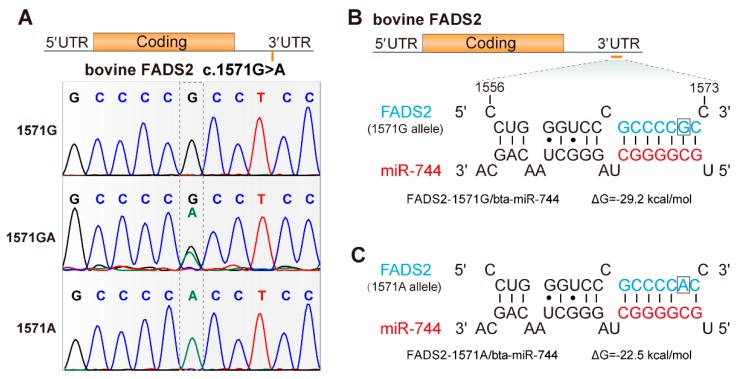
The minor A allele of SNP c.1571G>A may disrupt the ability of miR-744 to bind *FADS2*. (**A**) Sequencing map revealed SNP c.1571G>A situated in the 3′ UTR of the *FADS2* gene. (**B**,**C**) Computer alignment demonstrated SNP c.1571G>A altered a binding site for miR-744. Nucleotides of the miR-744 seed region (positions 2–8) are marked in red. The alleles are indicated in the box.

To confirm whether the potential functional SNP c.1571G>A existed in Chinese Holstein cows, we sequenced the 3′ UTR of bovine *FADS2* and aligned the sequence to the *Bos taurus* reference. As expected, SNP c.1571G>A was identified (Figure 1A). To evaluate the preliminary association of SNP c.1571G>A with FAs profiles, a population of 275 Chinese Holstein cows were genotyped using the Sequenom MassARRAY platform. The genotype and allele frequencies for the SNP c.1571G>A in *FADS2* are summarized in Table 1. The major allele frequency (c.1571G) was 0.844 and the population was in Hardy–Weinberg equilibrium (*χ*^2^ = 0.340).

### 3.2. Associations between c.1571G>A and Fatty Acids Profiles

To determine possible associations of *FADS2* c.1571G>A with milk FAs content, we analyzed the relationships between the *FADS2* genotype and FAs profiles in 275 Chinese Holstein cows. The analysis suggested that SNP c.1571G>A was significantly associated with different individual milk FAs (Table 2). In particular, compared with the GG genotype, genotype AA individuals had the higher value for C4:0 (*p* = 0.041), GLA (C18:3n-6, *p* < 0.001), and delta-6 desaturase activities as estimated by D6D index (C18:3n-6/C18:2n-6 ratio; *p* < 0.001), as well as the lower content for LA (C18:2n-6, *p* < 0.001, Table 2). These results were consistent with the allele substitution effects whereby substituting allele G for A increased milk LA by 0.541 and decreased milk GLA by 0.028 (Table 2). In addition, there was a significant association between c.1571G>A and the omega-6 LC-PUFA, dihomo-gamma-linolenic acid (DGLA, C20:3n-6; *p* = 0.004). In this case, the genotype GA was correlated with the highest increase in milk DGLA content (Table 2). These results indicated that SNP c.1571G>A was a functional marker with the c.1571A allele being associated with a higher *FADS2* delta-6 desaturase activity.

### 3.3. Effects of the SNP c.1571G>A on FADS2 Transcriptional Activity

The SNP c.1571G>A is located in the 3′ UTR of *FADS2* (Figure 1A). Recent evidence that a polymorphism in the 3′ UTR can perturb miRNA-directed inhibition of mRNA is of particular interest [27,28,29]. We therefore assessed the binding of miRNAs to *FADS2* mRNA in silico using RNA22 [23], RNAhybrid [24], and TargetScan software [25], and revealed that c.1571G>A polymorphism was perfectly complementary to the miR-744 seed region (Figure 1B), where miRNA–mRNA duplexes formed a complex with RISC (RNA-induced silencing complex), which is crucial for mRNA regulation. For each allele in SNP c.1571G>A, we calculated minimum free energy (mfe), which indicated the stability of miRNA–mRNA duplexes. A difference was observed, where the G>A substitution increased the predicted mfe from −29.2 to −22.5 kcal/moL (Figure 1B,C). This may potentially decrease the miR-744 binding affinity to the *FADS2* target region and increase the *FADS2* expression level in the presence of the A allele. These in silico data indicated that c.1571G>A could affect miR-744-related regulation of *FADS2* by changing its binding activity.

**Figure 2:**
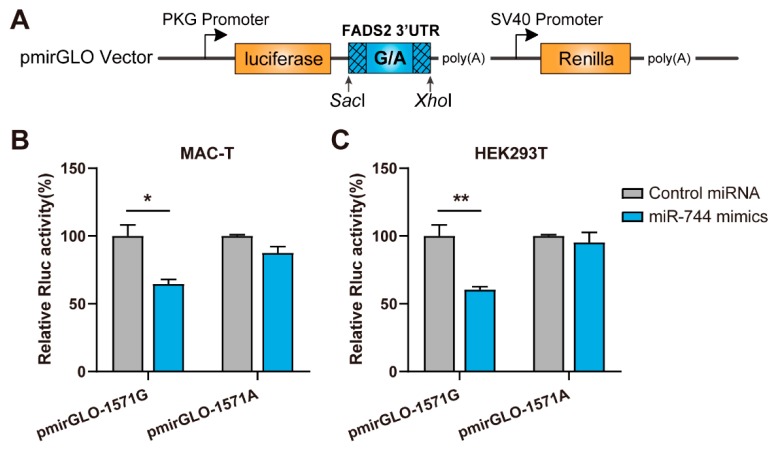
SNP c.1571G>A affects the binding of miR-744 to *FADS2*. (**A**) Schematic representation of the dual luciferase constructs harboring different alleles. (**B**,**C**) Relative luciferase activities are decreased in the presence of the G allele and miR-744, as compared with the A allele and miR-744, in MAC-T (**B**) and HEK293T cells (**C**). Error bars (SE) are derived from three independent experiments performed in triplicate, and data were compared using a Student’s *t*-test. * *p* < 0.05, ** *p* < 0.01.

In order to validate the computational prediction and to investigate the functionality of the SNP c.1571G>A, we next performed an in vitro luciferase assay. MAC-T and HEK293T cells were cotransfected with either pmirGLO-1571G or pmirGLO-1571A vectors (see methods) with either miR-744 mimics or a control miRNAs (Figure 2A). The effects of miR-744 mimics overexpression were verified by using RT-qPCR (Figure S1A). Consistent with our computational prediction, when the c.1571G allele variant was expressed in the presence of miR-744, there was a pronounced reduction in Firefly luciferase activity in both MAC-T (with a relative decrease of 35.3 ± 5.6%; *p* = 0.016) and HEK293T cells (with a relative decrease of 39.6 ± 3.8%, *p* = 0.010). On the contrary, cotransfection of the c.1571A allele variant and miR-744 did not result in a significantly different Firefly luciferase activity in either MAC-T or HEK293T cells (Figure 2B,C). These results validated that *FADS2* was a genuine target for miR-744. Moreover, these functional analyses demonstrated that the c.1571G allele resulted in significantly lower luciferase activity compared with the A allele, thus confirming that in vitro interaction between miR-744 and *FADS2* was hindered by SNP c.1571G>A.

### 3.4. Mir-744 Regulates FADS2 Expression in MAC-T cells

**Figure 3:**
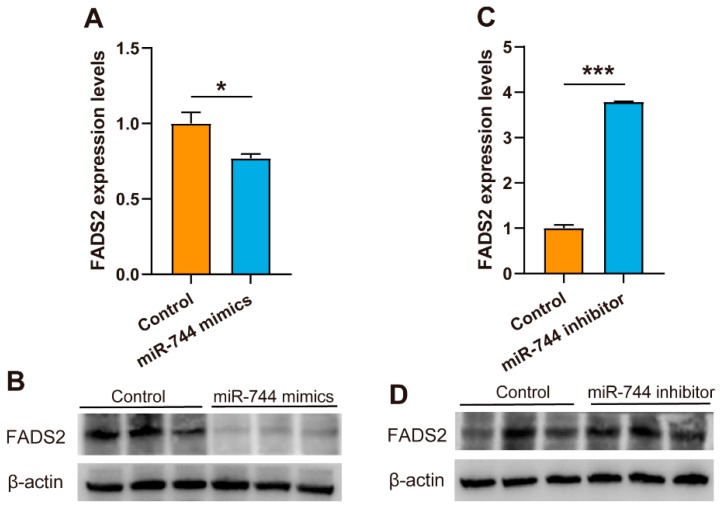
Influence of miR-744 on endogenous *FADS2* expression in MAC-T cells. (**A**–**D**) MAC-T cells were transfected with a control miRNA, miR-744 mimic, or miR-744 inhibitor as indicated. Twenty-four hours after transfection, the relative *FADS2* mRNA level was measured by RT-qPCR (**A**,**C**). Error bars (SE) are derived from three independent experiments performed in triplicate, and data were compared using a Student’s *t*-test. * *p* < 0.05, *** *p* < 0.001. Forty-eight hours after transfection, *FADS2* protein expression was examined by Western blot (**B**,**D**).

To further determine the miR-744/*FADS2* binding and to evaluate whether SNP c.1571G>A participated in the regulation of *FADS2* expression, we examined the regulatory effect of miR-744 on *FADS2* mRNA and protein expression levels in MAC-T cell line having *FADS2-1571GG* genotype. To do this, we overexpressed miR-744 levels using miR-744 mimics. Forty-eight hours post-transfection, cells overexpressed for miR-744 exhibited significantly lower *FADS2* mRNA levels compared with cells expressing the control miRNA (Figure 3A). Accordingly, declined protein expression of FADS2 was also detected in MAC-T cells overexpressing miR-744 (Figure 3B). In contrast, inhibition of endogenous miR-744 with a specific inhibitor dramatically downregulated miR-744 expression levels (Figure S1B) and upregulated *FADS2* expression at both mRNA and protein levels (Figure 3C,D). SNP c.1571G>A, therefore, resulted in a decrease of miR-744 binding to its target *FADS2*, with a consequent increase in *FADS2* expression levels.

## 4. Discussion

The *FADS2* gene, coding for delta-6 desaturase, is critical for the endogenous synthesis of LC-PUFAs from their precursor essential fatty acids, linoleic acid (LA, C18:2n-6) and α-linolenic acid (ALA, C18:3n-3), by a successive series of desaturation and chain elongation reactions. This gene is generally considered to be the rate-limiting enzyme in the biosynthesis of LC-PUFAs [30]. Therefore, exploiting animals’ inherent abilities by detecting *FADS2* gene mutations with improved delta-6 desaturase activity and incorporating them into breeding programs may result in an increase in milk LC-PUFAs content. In the current study, we revealed that SNP c.1571G>A may regulate *FADS2* expression by modifying miR-744 binding, and subsequently alter FAs composition in milk. Genotype distribution demonstrated that the c.1571G allele was more frequent (0.844) in Chinese Holstein cows. This value is a little higher than frequencies evaluated in a preceding study (0.844 vs. 0.731) [20]. This might be attributed to the different breeding direction in Chinese Holstein cows that favored the G allele, nonrandom mating, limited population size, or simply the greater number of farms involved in the preceding study.

Compared with the GG genotype, individuals carrying the AA genotype had a lower level of milk LA (C18:2n-6) and a higher level of GLA (C18:3n-6), indicating that the AA genotype is beneficial for the conversion of C18:2n-6 to C18:3n-6, which is the first step of the enzymatic cascade of n-6 LC-PUFA synthesis [31,32]. These results were also supported by the allele substitution effects; allele A, the minor allele with a frequency of 15.6% in the studied population, was superior in improving the proportion of GLA. The precursor/product FA ratios, referred to as the “desaturation indexes”, are usually used to estimate desaturase activities. For example, the ratio of C18:3n-6/C18:2n-6 is regularly used as a representative of the delta-6 desaturase activity (D6D index) of *FADS2* in genome-wide association studies (GWAS) and clinical studies [33]. In this respect, individuals carrying the AA genotype had increased D6D index, indicating that the effects of this SNP were possibly caused by decreasing the delta-6 desaturase activity of *FADS2*. Taken together, our results supported the use of allele A as a potential genetic marker to improve milk quality by increasing n-6 LC-PUFA content. However, considering the limited population size and milk samples used in the present study, the effects of c.1571G>A should be evaluated in future studies with larger subsamples.

Increasing evidence suggests that polymorphisms in the 3′ UTRs of genes are associated with complex economic traits by affecting miRNA-related regulation gene expression [34,35,36]. For example, Clop et al. demonstrated that a G-to-A transition in the 3′ UTR of the myostatin gene generated a binding site for miR-1 and miR-206, which inhibited the translation of the myostatin gene and hence contributed to the muscular hypertrophy of Texel sheep [34]. It is worth noting that the SNP c.1571 G>A occurred within the 3′ UTR of *FADS2*. This prompted us to determine the underlying metabolic mechanisms through which the SNP c.1571G>A polymorphism influenced FAs profiles. For this purpose, we evaluated the binding of miRNAs to *FADS2* mRNA in silico and revealed that c.1571G>A polymorphism was perfectly complementary to the miR-744 seed region, indicating that this SNP could affect miR-744-regulated *FADS2* expression by affecting its binding affinity.

Several studies have reported the transcriptional control of *FADS2* gene expression. For example, Ralston et al. described that treatment of differentiated 3T3-L1 adipocytes with ARA significantly downregulated the expression levels of *FADS2* [37]. Dong et al. reported that the promoter activities of *FADS2* were regulated by transcription factors SREBP1 and PPARα, two major regulators involved in fatty acid biosynthesis. SREBP1 and PPARα participated in the regulation of fatty acid metabolic genes expression via modulating *FADS2* expression [38]. Here, we illustrated the importance of post-transcriptional regulation of *FADS2* gene expression by miRNAs. Dual-luciferase reporter assay demonstrated that relative luciferase activity of G allele-corresponding constructs was significantly reduced in the presence of miR-744, while no such effect was observed with the A allele. Overexpression of miR-744 in MAC-T cells (having *FADS2-1571GG* genotype) downregulated *FADS2* expression at both mRNA and protein levels, whereas inhibition of endogenous miR-744 with a specific inhibitor increased *FADS2* expression. Together, these in silico and in vitro data indicated that SNP c.1571G>A abolished the ability of miR-744 to bind *FADS2*, leading to overexpression of its target and an increase in the synthesis of omega-6 PUFAs. These results are consistent with our findings that the A minor allele occurred with increased delta-6 desaturase activity of *FADS2* (as observed with C18:3n-6/C18:2n-6 ratio) and could at least partially account for the observed allele-dependent associations between c.1571 G>A and LA and GLA levels. However, the regulation of c.1571G>A on *FADS2* expression and fatty acid profiles in different tissues (e.g., liver, blood) and at different lactation periods should be investigated in future studies, as this may provide a deeper understanding of regulatory mechanisms of fatty acid metabolism.

## 5. Conclusions

In summary, the current study is the first to verify that SNP c.1571 G>A can alter *FADS2* expression and affect cow milk fatty acid composition through modifying miR-744 binding. SNP c.1571G>A, by disrupting the ability of miR-744 to bind *FADS2*, may contribute to the synthesis of omega-6 LC-PUFAs. Individuals carrying the AA genotype have a higher value for delta-6 desaturase activities, indicating favorable effects of the AA genotype on milk quality improvement. Therefore, *FADS2* c.1571 G>A is an interesting candidate for assisted selection programs to increase the healthier fatty acid composition of milk and, together with other reported causative SNPs, such as *DGAT1* K232A and *SCD1* A293V [39], may contribute to the improvement of bovine milk quality.

## Figures and Tables

**Table 1:** Allele, genotype frequency, and Hardy–Weinberg equilibrium test for the SNP c.1571G>A in the fatty acid desaturase 2 gene (*FADS2*) in Chinese Holstein cows.

SNP	Allele	Allele Frequency	Genotype	Genotype Frequency	Observed Count	Expected Count	χ^2^ Value of HWE Test	*p*-Value
*FADS2* c.1571G>A	G	0.844	GG	0.716	197	195.72	0.340	0.844
	A	0.156	GA	0.255	70	72.55		
			AA	0.029	8	6.72		

**Table 2:** Effects of SNP c.1571G>A on milk fatty acid-related traits of Chinese Holstein cows.

Fatty Acid-Related Traits ^1^	Genotype			Gene Effects ^2^			*F*	*p*-Value
	*GG*	*GA*	*AA*	*a*	*d*	*α*		
C4:0	3.311 ^ab^ ± 0.050	3.216 ^a^ ± 0.072	3.791 ^b^ ± 0.149	−0.240	−0.335	−0.471	3.237	0.041
C18:0	12.045 ^a^ ± 0.245	14.372 ^b^ ± 0.370	13.459 ^ab^ ± 0.822	−0.707	1.620	0.408	16.669	0.000
C18:2n-6	5.583 ^a^ ± 0.330	3.552 ^b^ ± 0.178	3.286 ^b^ ± 0.181	1.149	−0.883	0.541	14.068	0.000
C18:3n-6	0.364 ^a^ ± 0.012	0.465 ^b^ ± 0.013	0.480 ^b^ ± 0.042	−0.058	0.043	−0.028	21.087	0.000
C20:3n-6	0.112 ^a^ ± 0.007	0.147 ^b^ ± 0.012	0.134 ^ab^ ± 0.032	−0.011	0.024	0.006	5.694	0.004
SFA	65.835 ^a^ ± 0.465	68.988 ^b^ ± 0.618	67.35 ^ab^ ± 1.314	−0.758	2.395	0.890	3.384	0.000
D6D index	0.102 ^a^ ± 0.004	0.140 ^b^ ± 0.004	0.148 ^b^ ± 0.013	−0.023	0.015	−0.013	25.001	0.000

^a,b^ Means with different lowercase superscripts within a row differ (*p* < 0.05). ^1^ D6D index = 18:3n-6/18:2n-6. ^2^ *a* = allele additive effects, *d* = allele dominant effects, *α* = allele substitution effects.

## Supplementary Materials

The following are available online at https://www.mdpi.com/2076-2615/9/12/1090/s1, Figure S1: Efficiency of miR-744 inhibition and overexpression. Table S1: Descriptive statistics for milk fatty acids composition (g/100g of fatty acids). Table S2: Specific sequence used for SNP identification and RT-qPCR.
