# Supplementary material for: A Functional 3′ UTR Polymorphism of FADS2 Affects Cow Milk Composition through Modifying Mir-744 Binding
Source: Animals (Basel). 2019 Dec 6;9(12):1090. doi: 10.3390/ani9121090 (PMC6941027; doi:10.3390/ani9121090)
Supplement: Supplementary file 1 [file animals-09-01090-s001.pdf]

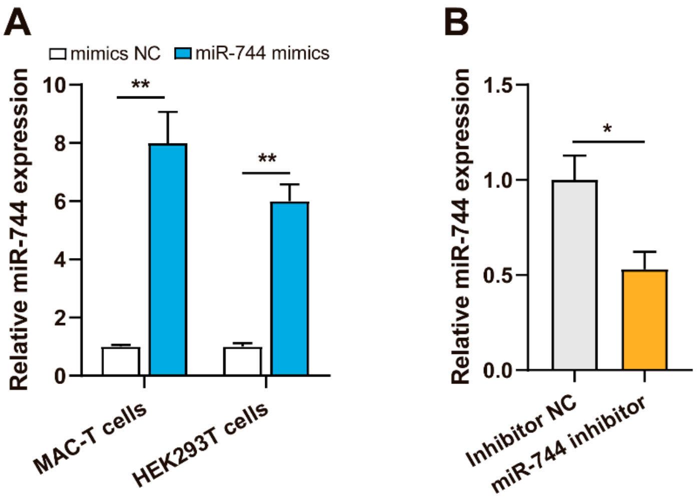

Figure S1. Efficiency of miR-744 inhibition and overexpression. MAC-T cells or HEK293T cells were transfected with a control miRNA, miR-744 mimic or miR-744 inhibitor as indicated. Twenty-four hours after transfection, the effects of miR-744 mimics overexpression (A) and the inhibitory efficiency of miR-744 inhibitor (B) were verified by RT-qPCR. Error bars (SE) are derived from three independent experiments performed in triplicate, and data were compared using a Student's *t*-test. * $P<0.05$, ** $P<0.01$.

Table S1. Descriptive statistics for milk fatty acids composition (g/100g of fatty acids)

Traits	Parity2		Parity3	
	Mean	SE	Mean	SE
C4:0	3.21	0.04	3.41	0.08
C6:0	1.94	0.03	2.17	0.07
C8:0	1.17	0.03	1.28	0.04
C10:0	2.63	0.06	2.85	0.10
C11:0	0.08	0.00	0.08	0.01
C12:0	3.05	0.08	3.28	0.11
C13:0	0.11	0.00	0.11	0.00
C14:0	9.81	0.16	10.08	0.26
C14:1	0.88	0.02	0.92	0.07
C15:0	1.14	0.02	1.10	0.04
C16:0	30.05	0.25	28.61	0.41
C16:1	1.55	0.10	1.49	0.13
C17:0	0.70	0.03	0.72	0.09
C17:1	0.21	0.01	0.20	0.02
C18:0	12.94	0.24	12.52	0.41
C18:1n9c	24.14	0.36	24.68	0.52
C18:2t	0.33	0.06	0.41	0.13
C18:2n-6	4.58	0.29	5.49	0.47
C18:3n-6	0.43	0.01	0.39	0.02
C20:0	0.12	0.01	0.15	0.01
C20:3n-6	0.14	0.01	0.14	0.01
C20:4	0.08	0.01	0.09	0.01
C22:6	0.02	0.01	0.06	0.02
D6D index[1]	0.12	0.00	0.11	0.01
SFA	67.03	0.45	66.18	0.73
MUFA	27.43	0.36	27.50	0.51
PUFA	5.53	0.28	6.32	0.45

[1]D6D index = 18:3n-6/18:2n-6

Table S2. Specific sequence used for SNP identification and RT-qPCR.

Sequence Definition	Product Length(bp)	Annealing temperature (℃)	Sense Primer	Antisense Primer	Use
FADS2	471	59	CGCCTACCTCCACAAATGAA	ACCCAGGGAATCCAGCAC	SNP detection
FADS2	131	60	GGTACCCAATGGGACAAAGA	TAGTGGGTTAATTTCCAGATGC	qRT-PCR
ACTB	100	60	TCTTCGCCTTAATACTTGT	AAGCCTTCATACATCTCAA	qRT-PCR
GAPDH	92	60	CTGAGAATCTCCTGACTT	TTATTGATGGTACACAAGG	qRT-PCR
bta-miR-744		60	GGGGCTAGGGCTAACAGCAA		qRT-PCR
U6		60	GGAACGATACAGAGAAGATTAGC	TGGAACGCTTCACGAATTTGCG	qRT-PCR
